# Exosomes from bulk and stem cells from human prostate cancer have a differential microRNA content that contributes cooperatively over local and pre-metastatic niche

**DOI:** 10.18632/oncotarget.6540

**Published:** 2015-12-09

**Authors:** Catherine A. Sánchez, Eliana I. Andahur, Rodrigo Valenzuela, Enrique A. Castellón, Juan A. Fullá, Christian G. Ramos, Juan C. Triviño

**Affiliations:** ^1^ Urology Department, Las Condes Clinic, Santiago, Chile; ^2^ Faculty of Science, University of Chile, Santiago, Chile; ^3^ Faculty of Medicine, University of Chile, Santiago, Chile; ^4^ Sistemas Genómicos, Valencia, España

**Keywords:** prostate cancer, miRNAs, exosomes, next generation sequencing, niche

## Abstract

The different prostate cancer (PCa) cell populations (bulk and cancer stem cells, CSCs) release exosomes that contain miRNAs that could modify the local or premetastatic niche. The analysis of the differential expression of miRNAs in exosomes allows evaluating the differential biological effect of both populations on the niche, and the identification of potential biomarkers and therapeutic targets. Five PCa primary cell cultures were established to originate bulk and CSCs cultures. From them, exosomes were purified by precipitation for miRNAs extraction to perform a comparative profile of miRNAs by next generation sequencing in an Illumina platform. 1839 miRNAs were identified in the exosomes. Of these 990 were known miRNAs, from which only 19 were significantly differentially expressed: 6 were overexpressed in CSCs and 13 in bulk cells exosomes. miR-100-5p and miR-21-5p were the most abundant miRNAs. Bioinformatics analysis indicated that differentially expressed miRNAs are highly related with PCa carcinogenesis, fibroblast proliferation, differentiation and migration, and angiogenesis. Besides, miRNAs from bulk cells affects osteoblast differentiation. Later, their effect was evaluated in normal prostate fibroblasts (WPMY-1) where transfection with miR-100-5p, miR-21-5p and miR-139-5p increased the expression of metalloproteinases (MMPs) -2, -9 and -13 and RANKL and fibroblast migration. The higher effect was achieved with miR21 transfection. As conclusion, miRNAs have a differential pattern between PCa bulk and CSCs exosomes that act collaboratively in PCa progression and metastasis. The most abundant miRNAs in PCa exosomes are interesting potential biomarkers and therapeutic targets.

## INTRODUCTION

Prostate cancer (PCa) is a major health concern, representing the second leading cause of cancer related death in men in developed countries, and the main cause of cancer death in elderly men. Early detection is fundamental to decrease its mortality [1, 2]. Screening is the main tool for early detection, although the protocols are continuously evolving to include multivariate tools calculator that include new diagnostic and prognostic markers [3]. The development of new diagnostic and predictive tools is a challenge that could be achieved by understanding key cancer-related pathways [4] for tumor growth and spread.

In PCa, it has been proposed that stem cells with cancer characteristics (therefore called cancer stem cells or CSCs) give rise and maintain tumor growth. These are a subset of cells into the tumor with abilities of self-renewal, giving rise to other malignant clones including transient amplifying cells (responsible for the bulk tumor proliferation) that repopulate the tumor after radio- and chemo-therapies and are responsible for development of castration-resistant disease [5-7]. Cancer cells also promote malignant progression by modifying the niche and surrounding cells, including fibroblasts. The recruitment of stromal fibroblasts, denominated cancer associated fibroblasts (CAFs) triggers matrix remodeling, and produces growth factors that promote tumor growth and progression. This communication is achieved among others, by the release of exosomes from cancer cells [8, 9].

Exosomes are vesicles released by cells that contain cytoplasmic content including several species of RNA, including miRNAs, that are delivered functionally into new cells modifying protein expression [10, 11]. miRNAs regulate gene expression, effect that is finally traduced in inhibition of the expression of groups of proteins related with common pathways. In cancer, altered expression of miRNAs is an important hallmark that can be detected in blood as changes in miRNA profiles and/or increase of their concentration [12].

Most of the miRNAs found in body fluids are included in exosomes [13], although some miRNAs can be found free in plasma associated to Ago [14, 15]. For this reason, the isolation of circulating exosomes could be a better method for miRNA screening in plasma and disease detection [12, 16]. Exosome isolation by minimal invasive procedures, makes them a great tool for description of new diagnostic markers [17], even their own circulating levels [12].

The miRNA profiles from cancer cells and their circulating exosomes, have shown strong differences [18], or similarities [12] depending on techniques used. miRNA expression is often analyzed by microarrays that limit the number of species analyzed to those miRNAs previously described in origin cells. The High-throughput sequencing by next generation sequencing (NGS) techniques improve profiling for gene expression, with a genome wide approach that increases the coverage of miRNAs, allowing the description of new species, an important difference respect to quantitative PCR (qPCR), the gold standard for transcript quantification [19]. Deep sequencing has several advantages over microarrays, mainly by overcoming the limitation of relative detection of previously reported miRNA sequences, and obtaining absolute abundance, allowing the detection of novel miRNAs [20], increasing the description and functional analysis of miRNAs, evaluating by quantitative analysis their expression levels, which are related with different stages of cancer initiation and progression [21].

Previously, we characterized a model that allowed us to study CSCs by an enrichment method from primary cultures where the amount of CSCs is limiting (around 1%) [22]. After obtaining PCa primary cultures where the bulk cells were the predominant population, cells were grow unattached in absence of fetal bovine serum (FBS). After 21 days we obtained prostatospheres enriched in around a 95% of CSCs with a CD44+/CD133+/ALDH+/ABCG2+/CD24- phenotype. Using this approach, we analyzed the profile of miRNAs secreted in exosomes from bulk cells and CSCs by NGS with Illumina Hiseq2000.

Our aim was to know the miRNAs differentially released by PCa cell populations and describe their effect in cancer related microenvironment changes and metastatic niche preparation by bioinformatics and functional assays over normal fibroblasts that lead to invasion and metastasis and the identification of potential diagnostic biomarkers and therapeutic targets.

## RESULTS

### miRNA expression in exosomes from PCa cells

**Figure 1:**
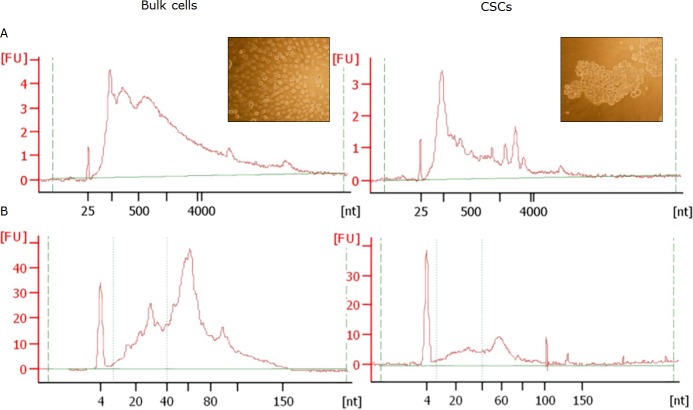
RNA profiles from exosomes from bulk cells (left) and CSCs (right) exosomes obtained by Agilent 2100 Bioanalyzer The electropherograms shows the size in distribution of the nucleotides (nt) and fluorescence intensity (FU) of Total RNA by Agilent RNA 6000 Pico Kit **(A)** and small RNAs after enrichment by the Agilent Small RNA Kit **(B)** All the electropherograms correspond to the sample of the same patient. The inserts show a representative image from the cultures under study.

Primary cell cultures from 5 PCa patients were obtained. These cultures were denominated bulk cells. From them, CSCs enriched prostatospheres (CSCs in ahead) were obtained (Figure 1, insert). Exosomes were isolated from culture media from bulk and CSCs cultures, and from them, total RNA was obtained. The RNA profile assessed by Bioanalyzer was similar between the 5 patients (Figure 1A). Small RNAs was the main RNA fraction observed in exosomes, mainly in CSCs exosomes. The ribosomal RNA peaks were small or absent in bulk cell exosomes (Figure 1A, left) and undetectable in CSCs exosomes (Figure 1A, right). Later, miRNA were isolated from total RNA, obtaining enriched fractions, mainly around 30 and 60 nucleotides (Figure 1B).

To compare the miRNA cargo of bulk and CSCs exosomes, we performed NGS. Samples were analyzed using the ultrasequencer Illumina HiSeq2000, a platform that delivers high yield of high quality data.

After quality controls and adapter elimination, only high quality reads were aligned using mirDeep2 algorithm against mirBase v20 database for the identification and quantification of previously described miRNAs. We identified 990 known miRNA species.

**Figure 2:**
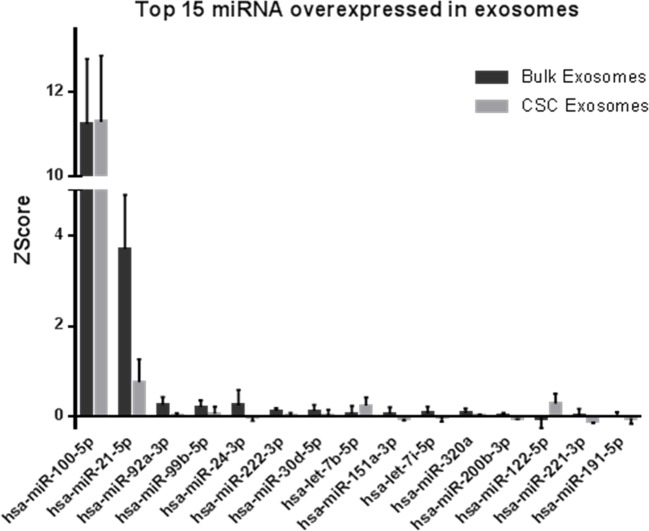
miRNAs abundance in exosomes from bulk and CSCs from PCa Relative expression expressed in ZScore of the top 15 miRNAs more abundant in exosome from bulk and CSCs from PCa cells (*n* = 5).

The total number of valid sequence reads was used as a measure of its relative abundance. Data were normalized by Z-score to compare relative expression in both samples (Figure 2).

Most of the miRNA species were detected in exosomes from both bulk and CSCs. From them, hsa-miR-100-5p and hsa-miR-21-5p were the most abundant miRNAs in exosomes from bulk and CSCs. hsa-miR-100-5p was expressed more than 10 times over the other miRNAs, with no differences at the exosome origin. On the other hand, hsa-miR-21-5p was highly expressed, but differentially over-expressed in bulk exosomes respect to CSCs.

Later, mirDeep2 was used for the prediction of novel miRNAs. This method uses the structure prediction by means of RNAfold to decrease the false positive ratio. Finally, these sequences were examined searching for cross-species conservation of the hairpin structure. Using this approach, 849 possible new miRNA were identified in the exosomes (Table 1).

**Table 1:** Top 10 new miRNAs in Bulk and CSCs exosomes

*Bulk Exosomes*			*CSC Exosomes*		
*microRNA ID*	*Mature Sequence*	*Similar microRNA in other organism*	*microRNA ID*	*Mature Sequence*	*Similar microRNA in other organism*
chr11:122022947	AACCCGUAGAUCCGAACUUGU	rno-miR-99a-5p	chr11:122022947	AACCCGUAGAUCCGAACUUGU	rno-miR-99a-5p
chr17:57918634	UAGCUUAUCAGACUGAUGUUGA	rno-miR-21-5p	chr17:57918634	UAGCUUAUCAGACUGAUGUUGA	rno-miR-21-5p
chr22:46508632	UGAGGUAGUAGGUUGUAUAGU	rno-let-7d-5p	chr22:46508632	UGAGGUAGUAGGUUGUAUAGU	rno-let-7d-5p
chr9:96938244	UGAGGUAGUAGGUUGUAUAGU	rno-let-7d-5p	chr9:96938244	UGAGGUAGUAGGUUGUAUAGU	rno-let-7d-5p
chr11:122017232	UGAGGUAGUAGGUUGUAUAGU	rno-let-7d-5p	chr18:56118320	UGGAGUGUGACAAUGGUGUU	rno-miR-122-5p
chrX:133303574	UAUUGCACUUGUCCCGGCCUG	rno-miR-25-3p	chr22:46509571	UGAGGUAGUAGGUUGUGUGGU	rno-let-7d-5p
chr9:96938635	UGAGGUAGUAGAUUGUAUAGU	rno-let-7d-5p	chr11:122017231	UGAGGUAGUAGGUUGUAUAGU	rno-let-7d-5p
chr13:92003578	UAUUGCACUUGUCCCGGCCUG	rno-miR-25-3p	chr7:25989542	UCAGUGCACUACAGAACUUUG	rno-miR-148b-3p
chr22:46509571	UGAGGUAGUAGGUUGUGUGGU	rno-let-7d-5p	chr11:122017232	UGAGGUAGUAGGUUGUAUAGU	rno-let-7d-5p
chr8:22102488	AAAAGCUGGGUUGAGAGGGCG	rno-miR-320-3p	chr11:122017230	UGAGGUAGUAGGUUGUAUAGU	rno-let-7d-5p

### Differential expression of miRNA between bulk and CSCs from PCa

**Figure 3:**
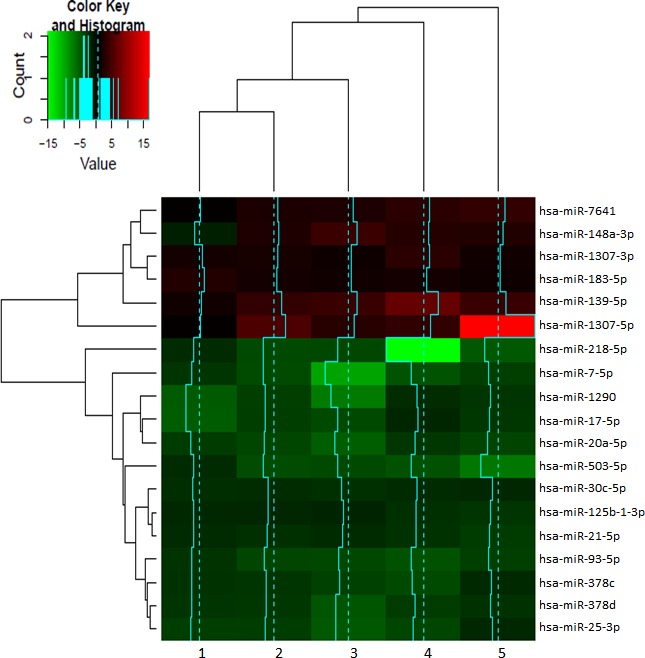
Heatmap of the 19 miRNAs differentially expressed in exosomes from CSCs respect to bulk cells The graph shows the miRNA expression from CSCs derived exosomes compared to the exosomes derived from its original bulk culture expressed in fold change. A complete linkage and Euclidean distance for the hierarchical clustering of samples and miRNAs was used (*n* = 5).

To identify the miRNAs differentially expressed between bulk and CSCs, the miRNAs with a fold change threshold of +/−2 and FDR adjusted p-value of 0.1 were selected. As result, 19 miRNAs were obtained (Figure 3). Six of them were overexpressed in CSCs exosomes: hsa-miR-7641, hsa-miR-148a, hsa-miR-1307-3p, hsa-miR-183, hsa-miR-139 and hsa-miR-1307-5p. Thirteen miRNAs were overexpressed in exosomes from bulk cells: hsa-miR-218-5p, hsa-miR-7-5p, hsa-miR-1290, hsa-miR-17-5p, hsa-miR-20a-5p, hsa-miR-503-5p, hsa-miR-30c-5p, hsa-miR-125b-1, hsa-miR-21-5p, hsa-miR-93-5p, hsa-miR-378c, hsa-miR-378d and hsa-miR-25-3p (Table 2).

**Table 2:** miRNAs differentially expressed in bulk (left) and CSCs (right) exosomes from PCa

*miRNAs overexpressed in Bulk exosomes*				*miRNAs overexpressed in CSCs exosomes*			
miRNAS	FoldChange	*p* value	padj	miRNAS	FoldChange	*p* value	padj
hsa-miR-7-5p	9.521	0.001	0.025	hsa-miR-1307-5p	11.180	0.009	0.092
hsa-miR-20a-5p	8.699	0.000	0.025	hsa-miR-139-5p	10.062	0.001	0.025
hsa-miR-93-5p	8.221	0.001	0.025	hsa-miR-148a-3p	5.281	0.012	0.092
hsa-miR-503-5p	7.823	0.013	0.097	hsa-miR-7641	4.933	0.014	0.097
hsa-miR-218-5p	7.759	0.009	0.092	hsa-miR-1307-3p	4.231	0.007	0.092
hsa-miR-1290	6.349	0.010	0.092	hsa-miR-183-5p	3.959	0.007	0.092
hsa-miR-25-3p	6.284	0.006	0.090				
hsa-miR-17-5p	6.139	0.010	0.092				
hsa-miR-378d	5.626	0.000	0.025				
hsa-miR-378c	5.009	0.002	0.025				
hsa-miR-21-5p	4.334	0.001	0.025				
hsa-miR-30c-5p	3.690	0.011	0.092				
hsa-miR-125b-1-3p	3.055	0.011	0.092				

### Validation of miRNAs in exosomes

**Figure 4:**
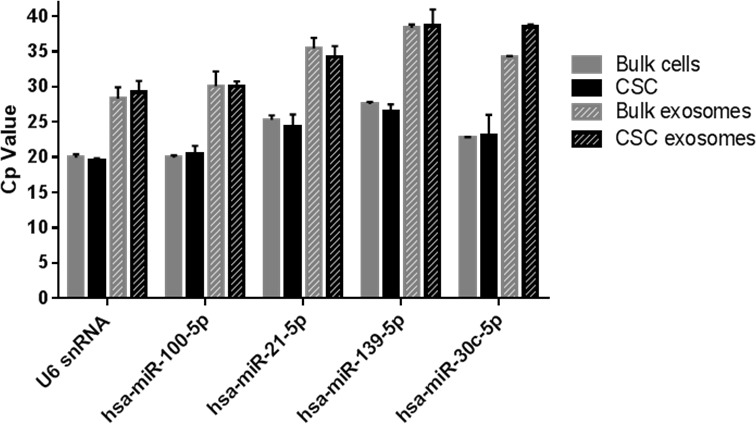
RT-qPCR on selected miRNAs in bulk and CSCs cells and exosomes Expression was assessed by TaqMan miRNA assays. Cp values from each assay are compared between samples (*n* = 3).

To validate the data obtained by NGS, the presence of the miRNAs sequenced was evaluated in exosomes and their origin cells by TaqMan miRNA Assays. The highly expressed hsa-miR-100-5p and hsa-miR-21-5p, and the differentially expressed hsa-miR-139-5p and hsa-miR-30c-5p, were detected in bulk cells and CSCs, and also in their exosomes. From these, hsa-miR-100-5p was the most abundant miRNA in all samples analyzed (Figure 4). U6 was detected for normalization and was present in all samples analyzed.

### Bioinformatics analysis of miRNAs in PCa exosomes

**Figure 5:**
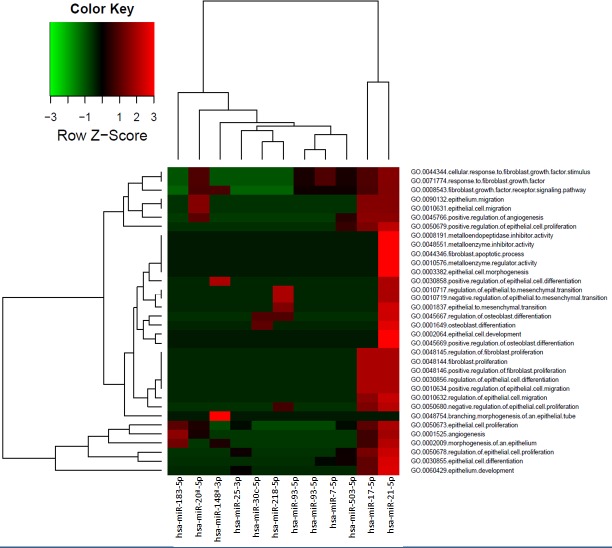
Biological process (GOBP) related with miRNAs differentially expressed in exosomes The color is related with the number of target genes (normalized by Z-Score) in each biological process analyzed.

The targets genes biologically annotated for the miRNAs differentially expressed in exosomes were identified using miRTarBase and TargetScan. Target genes were analyzed in function of biological processes related with prostate carcinogenesis, fibroblast activation and premetastatic niche modification (osteoblast differentiation) (Figure 5). In this analysis, the miRNAs with reliable and available annotation for genes targets and validated in laboratory experiments were included.

The main biological processes regulated by bulk exosomes miRNAs are the fibroblast growth factor (FGF) pathway, epithelial proliferation, differentiation and migration, and epithelial to mesenchymal transition (EMT) including activation of MMPs. Whereas for CSCs exosomes miRNAs the main functions are proliferation, differentiation to epithelium and angiogenesis. Most of the miRNAs modify the expression of several genes involved in a single molecular pathway. hsa-miR-21-5p and hsa-miR-17-5p have the strongest effect in the biological processes analyzed.

**Figure 6:**
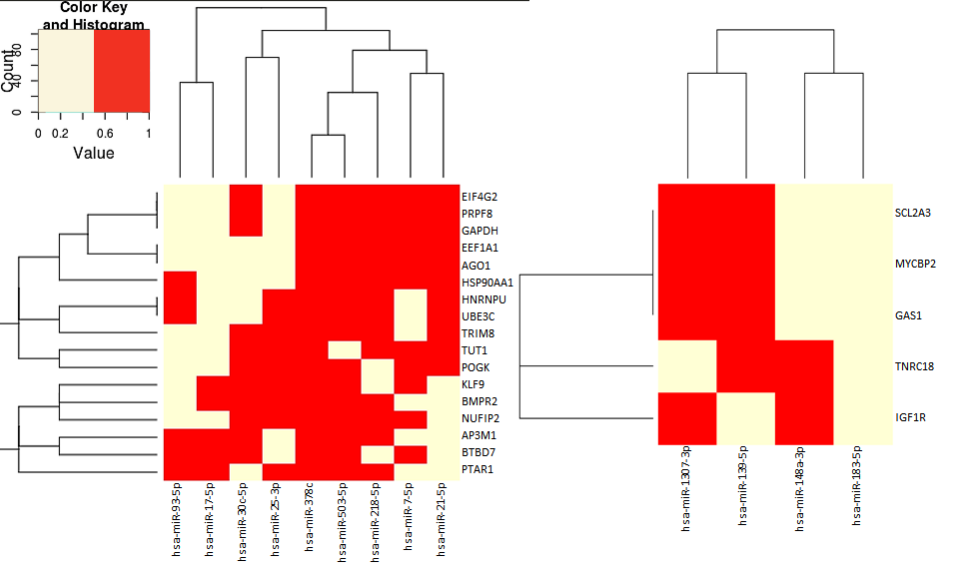
Target genes of miRNAs overexpressed in exosomes Targets analyzed separately for bulk cells (left) and CSCs (right) exosome miRNAs. Yellow = target of the miRNAs, Red = the gene is not target of the miRNAs.

Finally, to understand how the biological processes may be affected, the main common targets of the differentially expressed miRNAs from each population were analyzed (Figure 6). From bulk cell exosomes many miRNAs have a common set of targets, defining 3 clusters of miRNAs. From these targets, BMPR2 and HNRNPU are related with osteoblast differentiation that could be involved in the preparation of the premetastatic niche. Most of the other targets are related with regulation of the synthesis of proteins at different levels in transcription and translation and stability of the mRNAs.

The miRNAs that were differentially expressed in CSCs exosomes have, at this moment, less information in databases about their targets. Two clusters of miRNAs were defined according with their targets. These targets regulate glucose metabolism and proliferation, protein synthesis and degradation.

If not regulating directly, all the effects described for these miRNAs could have a consequence in the expression of proteins that coordinate changes in the biological processes described previously (Figure 5).

For the most expressed miRNA, hsa-miR100-5p, the bioinformatics analysis showed regulatory effects in cell proliferation, differentiation from epithelial cells and osteoblasts (Table 3).

**Table 3:** Functional analysis for hsa-miR-100-5p for selected biological processes

Biological Process	Description	Genes
GO:0050673	Epithelial cell proliferation	HMGN1,BMPR2,FGFR3,CDC73,DEAF1,RB1,MTOR,ATP5A1
GO:0001649	Osteoblast differentiation	RPS15,BMPR2,IARS,ID1,H3F3A,DDX21
GO:0050680	Negative regulation of epithelial cell proliferation	FGFR3,CDC73,RB1,ATP5A1
GO:0050678	Regulation of epithelial cell proliferation	HMGN1,BMPR2,FGFR3,CDC73,DEAF1,RB1,MTOR,ATP5A1

### Functional assay in normal fibroblasts

Cancer cells recruit stromal fibroblasts modifying their biological properties, leading to changes in the tumor microenvironment that favor progression. The effects of miRNAs from exosomes described by bioinformatics analysis that may be related with these functional changes were evaluated in a normal prostate fibroblasts cell line (WPMY-1). Cells were transfected by separated with 25 μM of miR100-5p (overexpressed in all exosomes), miR21-5p (overexpressed in bulk cell exosomes) and miR139-5p (overexpressed in CSCs exosomes). The miRNA let 7c was included as positive control for transfection. After transfection, the effect in the expression of proteins related with extracellular matrix restructuring (MMP-2, -9 and -13) and osteoclast recruitment and cell migration (RANKL) was evaluated.

**Figure 7:**
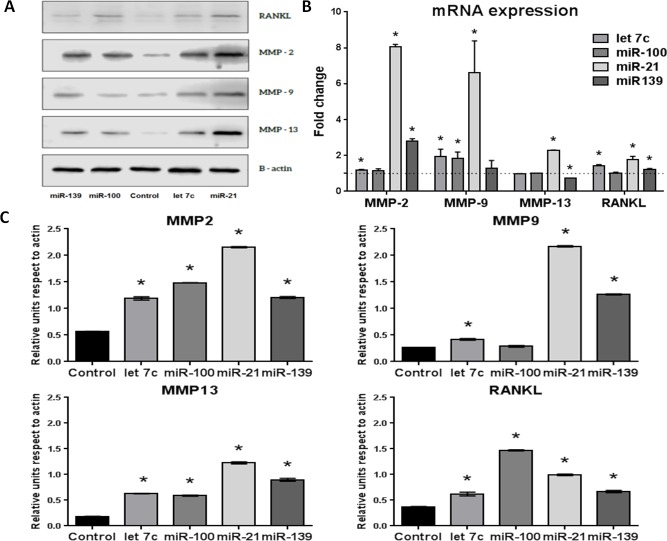
Effect of miRNA-100, -21, -139 and let 7c transfection of WPMY-1 cells on MMP-2, -9 and -13 and RANKL expression at 48 hours post-transfection **A.** Representative western blot of transfected cells. **B.** Fold change of mRNA expression in transfected cells evaluated by qPCR with the ΔΔCt method normalized by GAPDH. Dotted line represents the level of expression in non-transfected cells. **C.** Expression of proteins in transfected cells evaluated by western blot, normalized by actin. *n* = 3, *p < 0.05.

miRNAs increased significantly the expression of MMPs, mainly at protein level (Figure 7A and 7C). This effect was also observed at mRNA level (Figure 7B). miR-21 had the most significant effect over the expression of all MMPs, mainly up-regulating MMP-9 expression. miR-100 also increased MMP-2 and -13 but it had no effect on MMP-9. miR-139 also regulated the expression of all MMPs.

At the premetastatic niche, the microenvironment can be modified by changes in balance RANKL/OPG. RANKL secreted by osteoblasts and by metastatic PCa cells promotes osteoclast recruitment and differentiation, initiating the bone vicious circle. Besides, RANKL is secreted in the prostatic tumor, increasing levels of soluble RANKL and promoting cell migration. Transfection with miR-100, miR-21 and miR-139 increased significantly the expression of RANKL in fibroblasts at protein (Figure 7A and 7C) and mRNA levels (Figure 7B) that could act as a paracrine factor for cancer cells.

The changes in protein expression triggered by miRNAs were similar to the functional effects described by bioinformatics analysis. MMPs and RANKL overexpression induced by miRNAs in fibroblasts are related with cancer invasion and metastasis.

### Fibroblast migration

**Figure 8:**
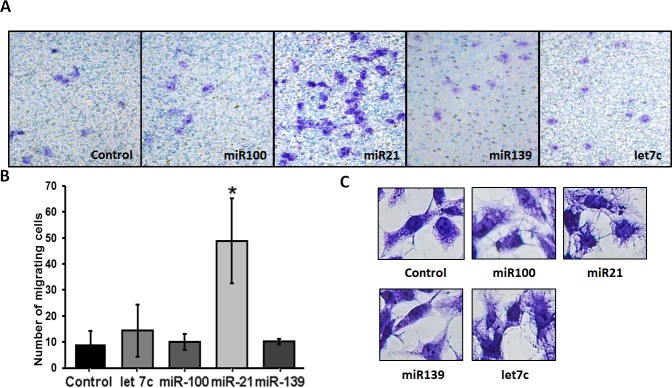
Effect of miRNA-100, -21, -139 and let 7c transfection on migration of WPMY-1 cells Five thousand transfected cells were seeded into each transwell and migration was evaluated after 24 hours. **A.** Representative images of migration assay at 24 hours. **B.** Number of migrating cells per field. *N* = 3, bars represent standard deviation, *p < 0.05 compared to control (non-transfected cells). **C.** Representative images of transfected WPMY-1 cells.

The recruitment of fibroblasts triggered by cancer cells exosomes, among others mechanisms, increases their migration ability. The effect of miRNAs from PCa exosomes in the migration of normal prostate fibroblasts was evaluated after 48 hours from transfection. miR-100, miR-21 and miR-139 increased the number of migrating cells when compared to control. However, only miR-21 increased significantly cell migration (Figure 8A and 8B). The increase in migration could be explained by morphological changes in transfected fibroblasts. miR-100 and miR-21 increased the number of prolongations when compared with miR-139 and non-transfected cells (Figure 8C).

## DISCUSSION

Tumors harbor a complexity of cell types that collaborate with survival and growth. The main population is constituted by clones of bulk cells, and a small population (near to 1%) are CSCs [23]. These populations interact between them promoting EMT and tumor progression [24], and with non-tumoral elements of the microenvironment, that collaborate with the growth and progression of the tumor [25, 26]. This communication is mediated, among others mechanisms, by exosomes that harbor a differential effect depending on the cell origin. Grange et al. demonstrated that microvesicles released by CSCs have a proangiogenic effect that is not observed in bulk microvesicles from renal cancer cells [27].

We observed that CSCs and bulk cells exosomes have a differential miRNA content that could trigger a differential effect over microenvironment. 990 known miRNAs were identified, most of them common. The most abundant, miR-100-5p and miR-21-5p were previously described in the top 10 miRNAs in exosomes from human esophageal cancer cells [28]. Bioinformatics analysis identified 19 miRNAs differentially expressed, including overexpression of miR-183 in CSCs and miR-7 in bulk cell exosomes that were previously described in human renal cancer [27].

From differentially expressed, miR-21-5p is the most abundant, even in all exosomes. miR-21-5p is a well described oncomir with potential clinical applications. In human ovarian cancer, miR-21 was isolated in tumor and peripheral blood-derived exosomes with a strong correlation [18]. In a meta-analysis, Wang et al. described that circulating miR-21 could significantly predict poorer survival in general carcinomas [29]. In prostate, miR-21 is overexpressed in cancer compared to normal cells, and it has a demonstrated role in carcinogenesis. Although its expression responds to androgen receptor (AR), miR-21 is overexpressed in AR negative PCa cells, increasing cell proliferation and triggering androgen independent growth, being sufficient to develop castration resistance [30]. miR-21 expression is related with histologic stage and biochemical recurrence [31] and with facilitating EMT by increasing expression of matrix remodeling proteins [32]. In our study, miR-21 increased expression of MMP-2, MMP-9 and MMP-13 in normal fibroblasts and triggered cellular changes related with fibroblast activation, as changes in the shape and increased migration. For these functions, miR-21 is considered an interesting therapeutic target.

hsa-miR100-5p was the most abundant miRNA in PCa exosomes. miR-100 has been related with prostatic carcinogenesis with a controversial role. In several studies it has been described as tumor suppressor, because its expression decreases in advanced PCa [33, 34]. However, its function could be context-dependent. miR-100, together with miR-let7c and miR-218 is significantly overexpressed in localized PCa (similar to the type of cells that we analyzed in our study) when compared with metastatic carcinoma [35].

One feature of the solid tumor cells that promote progression and recurrence after treatment is the ability to recruit surrounding stromal cells. These cells contribute to modify the microenvironment and promote angiogenesis by changing the expression of extracellular matrix proteins (e.g. collagen, fibrin and proteoglycans) and proteins that modify it (e.g. MMPs), facilitating invasiveness and metastasis [36]. The miRNAs overexpressed in bulk exosomes modify surrounding cells, regulating cell differentiation, proliferation, migration and EMT. In our study, we confirmed that miRNAs from PCa exosomes trigger overexpression of MMP-2, -9 and -13 and increased the migration in normal fibroblasts.

In behalf of the premetastatic niche preparation, miR-21, miR-30 and miR-218 regulate osteoblast differentiation, increasing the production of RANKL, among other soluble factors. This is a key step in the initiation of the bone vicious cycle that activates osteoclastogenesis [37]. PCa cells trigger changes in bone microenvironment by releasing RANKL at the metastasis. However, RANKL also plays a role at the primary tumor niche. RANKL is overexpressed by PCa cells increasing its level in the tissue [38], favoring progression by enhancing cancer cells migration and by modulating the immune response [39, 40]. According to our results, expression of RANKL in fibroblasts is increased by miRNAs from PCa exosomes, augmenting local levels of RANKL and making a favorable microenvironment for tumor progression. This could have an effect at distance increasing RANKL at the premetastatic niche, due to high local levels of RANKL could increase systemic levels, and by the release of exosomes, PCa cells could increase RANKL synthesis at the bone.

The miRNas that were differentially expressed in CSCs exosomes mainly regulate angiogenesis and cell proliferation. These functions are fundamental to promote tumor growth, survival and dispersion to distant niches. Grange et al. described previously that CSCs exosomes prepare the premetastatic niche in renal cancer, by overexpression, among others of miR-183 [27], that was also differentially expressed in PCa CSCs exosomes. The functions of differentially expressed miRNAs in exosomes from bulk and CSCs show a collaborative effect between these populations on PCa progression and invasiveness.

The bioinformatics analysis used included only miRNAs that are experimentally validated giving the power to predict functional targets that can be further validated. mirTarBase and TargetScan have been widely used in works that analyzed different tissues and diseases [41-43] and also been validated in prostate tumors [44-47]. Bioinformatics analysis also allows obtaining information about the “functional distance” between differentially expressed miRNAs. This makes possible to validate the importance of single miRNAs in each biological process, specifically is to propose the synergy between miR-21-5p and miR-7p in the regulation of prostate carcinogenesis. This collaboration is observed in genes regulated by two or more differentially expressed miRNAs, e.g. EEF1A1, related with the progression and transformation of PCa [48], AGO1, related with miRNA mediated gene silencing in cancer [49] and KLF9, a transcriptional factor that inhibits AKT activation and inhibit the PCa cells growth [50].

The cell model and the methodology used to describe the miRNA profile are critical when comparing with previous data. Using high-throughput sequencing, Liao et al. (2014) described a pattern of miRNAs in human esophageal cancer cells exosomes similar to ours, while the profile of exosomes from the PCa cell line PC3 performed by microarray, the coincidences decreased significantly[16, 28]. NGS improves significantly the number of miRNAs identified in the samples increasing the detection and leading to the description of low expressed and new miRNAs [51].

Previous studies that have analyzed circulating miRNAs as potential candidates for PCa biomarkers identified miRNAs differentially expressed in bulk cell exosomes in our study, in particular mir-21-5p [52-54], miR-378c [55], miR-1290 [56], miR-20a [54, 57], miR-125b [58], miR-93-5p [59]. Exosomes are an excellent source of miRNAs [12, 18], especially when analyzing plasma samples, where the amount of miRNAs is at least 6 times higher inside the exosomes than free in plasma [13]. Besides, in PCa patients, the amount of circulating exosomes is higher than in normal patients [60]. This makes us think that most of the miRNAs that are increased in blood could be product of the exosomes released by bulk cells, the main cellular component of prostatic tumors, and could be a potential source of biomarkers of PCa, a disease that lacks of specific diagnostic markers.

As conclusion, this study point out that differentially expressed miRNAs in exosomes from PCa populations promote collaboratively microenvironment changes related to tumor growth and progression, and at distance, modify the premetastatic niche, suggesting a potential role as therapeutic targets. In the other hand, miRNAs overexpressed in bulk cells exosomes have been described previously as highly expressed in blood of patients with PCa and could be studied as potential biomarkers. Further additional functional studies will confirm our conclusions.

## MATERIALS AND METHODS

### Primary cell cultures and CSCs enriched prostatospheres

Primary cell cultures were established from prostate tissue from 5 patients with localized PCa (Gleason Score 5-6) to obtain adherent cells (bulk) [61]. Briefly, samples were cut into small pieces and enzymatically digested in a collagenase solution (collagenase, 2.5 mg/ml; hyaluronidase, 1 mg/ml; deoxyribonuclease, 0.01 mg/ml) for 2-3 h at 37°C in shaking water bath. The resultant large epithelial cell aggregates were isolated and further digested for another 8-12 h. Later, the small aggregates were mechanically dispersed, and grown in DMEM-F12 medium (Lifetechnologies) supplemented with 7% FBS (Cellgro, Corning) at 37°C with 5% CO_2_.

Prostate CSCs were isolated from primary cultures after amplification according to the protocol previously described [7, 22]. Briefly, after 3 passages of the cultures described above, cells were detached and cultured in non-adherent conditions in absence of FBS and with B-27 supplement (Gibco). Resulting tumorspheres were maintained during 2 weeks and evaluated for stem markers (CD44+/CD133+/ALDH+/ABCG2+/CD24-) therefore considered as CSCs. Later, the cells were plated at a density of 4×10^4^ cells/ml, in DMEM medium without FBS and supplemented with the following factors: 5 ug/ml of human transferrin, 5 ug/ml of insulin, 20 ng/ml of EGF, 10 ng/ml of FGF-2, 200 ng/ml of retinol, 200 ng/ml of vitamin E, 10 nM of hydrocortisone, 2 ng/ml of sodium selenite and 0.4% of human serum albumin-free globulins. This non-adherent culture medium allowed the formation of prostate spheroids (prostatospheres) enriched in CSCs. All protocols for PCa samples were approved by the institutional committee of bioethics.

### Cell line culture

Human prostatic normal fibroblast cell line WPMY-1 (CRL-2854) was obtained from American Type Culture Collection (Virginia, USA). WPMY cells were maintained in DMEM (Lifetechnologies) supplemented with 5% FBS at 37°C with 5% CO_2_.

### Exosome isolation

For bulk cells, cultures were grown until reach a 60% confluence. Then, cells were grown during 48 hours in DMEM-F12 supplemented with 5% exosome-depleted FBS (Exo-FBS, SBI). Later, culture medium was recovered and centrifuged at 2000 x g for 5 min, to eliminate cells, apoptotic bodies and debris, and then at 16500 x g for 20 min at 4°C to eliminate microvesicles [62]. For prostatospheres cultures, serum free culture medium was recovered after 21 days of growth and centrifuged at 2000 x g for 5 min and later the supernatant at 16500 x g for 20 min at 4°C. Later, exosomes were isolated by precipitation with the reagent ExoQuick-TC (SBI), according to manufacturer's instructions. Briefly, 10 ml of culture medium was mixed with 2 ml of ExoQuick-TC and incubated overnight at 4°C. Later, the mix was centrifuged at 1500 x g for 30 min at room temperature to obtain an exosome pellet.

### miRNA extraction

miRNA extraction from exosome pellet was performed with mirVana miRNA isolation kit (Ambion) according to manufacturer instructions in two steps process. First, exosomes were lysed and after organic extraction, total RNA was isolated over glass-fiber filter and eluted. The quality of the total RNA was assessed by Agilent 2100 Bioanalyzer using an RNA 6000 Pico Kit (Agilent Technologies). After verifying the quality of RNA, we proceeded to the second part of the protocol of small RNA enrichment from total RNA by increasing ethanol content of the sample and isolation over glass-fiber filter and elution. Later, quality of the small RNA samples was analyzed by Agilent 2100 Bioanalyzer using a Small RNA kit (Agilent Technologies). RNA total concentration was measured by Nanodrop, and miRNA concentration was measured analyzed with the Quant-iT RiboGreen Kit (Invitrogen).

### Next generation sequencing

NGS was performed in the Illumina platform (MiSeq, Illumina) at Sistemas Genómicos (Valencia, Spain). Briefly, the procedure included:

- Quality control of RNA: The quality and the quantity of the RNA were determined in Bioanalyzer 2100 Small RNA assay and Qubit 2.0 fluorometer.
- Libraries preparation: cDNA libraries were obtained following Illumina's recommendations. Briefly, 3′ and 5′adaptors were sequentially ligated to the RNA prior to reverse transcription and cDNA generation. The cDNA was enriched with PCR to create the indexed double stranded cDNA library. Size selection was performed using 6% polyacrylamide gel. The quality of the libraries was analyzed in Bioanalyzer 2100, High Sensitivity assay, and the quantity of the libraries was determined by real-time PCR in Light Cycler 480 (Roche).
- Prior to clusters generation in cbot (Illumina), an equimolar pooling of the libraries was performed. The pool of the cDNA libraries was sequenced by paired-end sequencing (100 × 2) in Illumina HiSeq 2000 sequencer.

### Bioinformatics analysis

Bioinformatics analysis was carried out by Sistemas Genómicos (Valencia, Spain). At primary level, the quality of the data was analyzed by fastQC program [63]. The low quality raw reads and technical adapter were filtered using Trim Galore [64]. Then the high quality reads ware mapped against the GRch37/hg19 Homo sapiens Genome, against the human mature miRNAs and hairpins present in the of mirBase v20 database [65] using Bowtie2 algorithms [66]. In this mapping process a seed length of 16 nucleotides was defined and a maximum of two mismatches were allowed in this seed.

For the identification and quantification of novel and known miRNAs, the miRDeep2 algorithm [67] and BedTools [68] were applied. In the novel miRNAs identification, mirDeep2 algorithm uses a structural prediction by means of RNAfold [69].

For differential expression between samples, the algorithm DESeq2 was applied [70] and a FoldChange threshold of 2 and pValue adjusted by FDR of 0.01 were selected for miRNAs differentially expressed between conditions.

For the identification of the targets genes biologically annotated of miRNAs differentially expressed, the miRTarBase [71]and TargetScan [72] were used. Finally, for the new targets genes prediction the mirMap bioinformatics software [73] was utilized with a pValue threshold of 0.01.

### Taqman miRNA assay

cDNA was synthetized from miRNA samples (300 ng) from exosomes and cells with the TaqMan^®^ MicroRNA Reverse Transcription Kit (Lifetechnologies) using a pool of primers included in the TaqMan miRNA assays for hsa-miR-100, hsa-miR-21-5p, hsa-miR-139-5p, hsa-miR-30c and U6 snRNA according to manufacturer's instructions. Later, cDNA was used to perform qPCR using the TaqMan^®^ Universal Master Mix II no UNG (Lifetechnologies) with primers included in the TaqMan miRNA assay according to manufacturer's protocols in a Light Cycler 480 thermocycler (Roche).

### miRNA transfection

Cells were grown until reach a 60-70% confluence. Then, cells were transfected separately with the miRNAs miR-100, miR-21 and miR-139. The miRNA let7c was included as a positive control of transfection. Cells were transfected according to the manufacturer's protocol. Briefly, cells were incubated with a mix of Opti-MEM medium (Lifetechnologies), Lipofectamine RNAi max (Lifetechnologies) and miRNA mimics (mirVana mimics, Life Technologies) to a final concentration of 20 nM. After 48 hours, total RNA and proteins from transfected and control cultures were extracted with the mirVana PARIS kit (Ambion) according to manufacturer's protocol. All transfections were performed in triplicate.

### Western blot

Whole cell protein lysates (25 μg) were electrophoresed on SDS-polyacrylamide gels, transferred to nitrocellulose membranes and probed with primary antibodies against MMP-2 and RANKL (sc-58386 and -377079, respectively, Santa Cruz Biotechnology), MMP-9 (D603H, Cell Signaling), MMP-13 (MAB511, R&D systems), and actin (MAB1501, Millipore). Goat anti-rabbit or goat anti-mouse IgG conjugated to horseradish peroxidase (Jackson ImmunoResearch) were used as secondary antibodies. Quantitative evaluation was performed by densitometry using the Scanner C-DiGit (LI-COR) and the software Image Studio for C-Digit. Intensities from each band were normalized to actin. Statistical analysis among transfected groups was made by U Mann Whitney test, considering a significance of *p* < 0.05.

### qPCR

cDNA was synthetized from 2 ug of total RNA extracted from transfected and control cells by reverse transcription with the SuperScript II Reverse Transcriptase system (Invitrogen) according to manufacturer's protocol. qPCR was performed in a Light Cycler 480 (Roche) thermocycler using specific primers for MMP-2, 3′ CCA CGT GAC AAG CCC ATG GGG CCC C 5′ *(fwd)*, 3′ GCA GCC TAG CCA GTC GGA TTT GAT G 5′ *(rev)*, MMP-9, 3′ CGC TGG GCT TAG ATC ATT CC 5′ *(fwd)*, 3′ TTG TCG GCG ATA AGG AAG G 5′ *(rev)* and MPP-13, 3′ TTG AGC TGG ACT CAT TGT CG 5′ *(fwd)*, 3′ GGA GCC TCT CAG TCA TGG AG 5′ *(rev)*, RANKL 3′ TGA TTC ATG TAG GAG AAT TAA ACA GG 5′ (*fwd)*, 3′ GAT GTG CTG TGA TCC AAC GA 5′ *(rev)* and GAPDH 3′ TGC ACC ACC TGC TTA GC 5′ *(fwd)*,3′ GGC ATG GAC TGT GGT CAT GAG 5′ *(rev)* that was included as control. All qPCR reactions were performed at 60°C. Quantification was performed using the method ΔΔCt respect to GAPDH.

### Cell migration

Migration of fibroblasts was evaluated using 24-well Transwell plates with 8-μm pore size polycarbonate membrane (Costar, Corning). After 24 hours of transfection with miRNAs, cells were harvested and suspended in DMEM containing 0.1% FBS. 5×10^3^ cells were loaded into each of the upper wells. The lower wells were loaded with DMEM supplemented with 10% FBS as chemotactic factor. Cells were incubated at 37°C for 24 h and then were fixed with methanol and stained with crystal violet (1%, w/v). Cells in the upper surface of the filter were removed, and migrating cells were visualized by microscopy. Images were captured and quantified by counting cells that migrated to the lower side on 5 random fields of the filter at low magnification (X200).
